# Exploring the Antiproliferative Activity of Flavolignans From the Leaves of *Casearia arborea* (Salicaceae)

**DOI:** 10.1002/cbdv.202501489

**Published:** 2025-09-05

**Authors:** Augusto L. Santos, Mariana T. Rodrigues, Ana Paula Michelli, Rodrigo E. Tamura, Ileana G. S. de Rubió, Marisi G. Soares, Marcelo J. P. Ferreira, Patricia Sartorelli

**Affiliations:** ^1^ Institute of Environmental, Chemical and Pharmaceutical Sciences Federal University of São Paulo São Paulo Brazil; ^2^ Programa De Pós‐Graduação em Biologia Estrutural e Funcional Federal University of São Paulo São Paulo Brazil; ^3^ Institute of Environmental, Chemical and Pharmaceutical Sciences Federal University of São Paulo São Paulo Brazil; ^4^ Institute of Chemistry Federal University of Alfenas Alfenas Minas Gerais Brazil; ^5^ Botany Department, Institute of Biosciences University of São Paulo São Paulo Brazil

**Keywords:** cytotoxic activity | flavolignan | molecular networking | thyroid cancer | tricin

## Abstract

The *Casearia* genus (Salicaceae) is well known as a source of natural antitumor prototypes, as described for clerodane diterpenes. In this way, the dichloromethane phase from leaves of *Casearia arborea* was phytochemically evaluated in liquid chromatography coupled to high‐resolution mass spectrometry, furnishing flavonoid, flavolignan, and phenylpropanoid derivatives, and apocarotenoid terpenoids according to Global Natural Products Social Molecular Networking (GNPS) dereplication. The flavolignans were chemically monitored by liquid chromatography using a UV–Vis detector and purified by a semi‐preparative liquid chromatographic system, furnishing tricin, tricin‐4″‐*O*‐(*threo*‐guaiacylglyceryl) ether (salcolin A), and tricin‐4″‐*O*‐(*erythro*‐guaiacylglyceryl) ether (salcolin B). Additionally, it was observed that tricin was able to reduce live cells and increase apoptotic cell lines of papillary thyroid cancer and induced only necrosis in this HTH83 cell line, whereas salcolins A and B induced cell death in anaplastic thyroid cancer, increasing cell necrosis. Still, there is no significant induction of apoptotic cell death or necrosis in papillary thyroid cancer cells.

## Graphical abstract

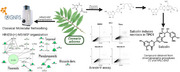

**Abbreviations**
ATC: anaplastic thyroid cancer
CMN: classical molecular networking
ESI: electron spray ionization
GNPS: Global Natural Products Social Molecular Networking
HPLC: high‐performance liquid chromatography
HRMS/MS: high‐resolution mass spectrometry coupled to mass spectrometry
LC‐DAD: liquid chromatography coupled to diode‐array detector
PTC: papillary thyroid cancer
QTOF: quadrupole coupled to time‐of‐flight mass analyzer
RP: reverse phase
UPLC–HRMS: ultra‐performance liquid chromatography coupled to high‐resolution mass spectrometry

## 1 Introduction

Thyroid cancer incidence is rising, accounting for 3% of all diagnosed cancers [1, 2]. Papillary thyroid carcinoma (PTC) is the most common type, making up about 80% of cases and with a 5‐year survival rate higher than 90% [3]. Although anaplastic thyroid cancer (ATC) is rare (1%–2% of cases), its aggressiveness accounts for one third of thyroid cancer deaths, with around 50% of patients having metastatic disease at the first diagnosis, and 25% developing metastasis and rapid disease progression [4]. ATC patients do not respond to radioiodine or chemotherapy, with a survival of 3–9 months after diagnosis [5].

Plants serve as sources for discovering new prototypes with antitumor potential because they contain a wide range of bioactive secondary metabolites [6]. In this context, Brazil has the highest biodiversity of plants in the world, including many medicinal species, and the Atlantic Forest is a conservation hotspot due to its high number of endemic species, making it one of the world's most important biomes [7, 8, 9]. The genus *Casearia* Jacq. (Salicaceae) includes roughly 217–277 species of shrubs and trees [10, 11, 12] found across the pantropical region, including Africa, Asia, Australia, the Americas, and Pacific islands [10, 13]. In Brazil, about 50 species of *Casearia* occur in nearly all biomes, with 24 being endemic [11, 14, 15]. The pharmacological properties of *Casearia* are varied, and researchers have been investigating secondary metabolites from 35 different *Casearia* species [16, 17, 18, 19, 20, 21]. Regarding chemical composition, clerodane diterpenes are chemical markers of *Casearia*, demonstrating high cytotoxic activity against various cancer cell lines, as shown by several authors in the review by Xia et al. [18]. Recently, a variety of flavonoids has been identified in different *Casearia* species, including *C. arborea* [20, 22, 23, 24], *C. decandra* [20], *C. gossypiosperma* [19], *C. grandiflora, C. javitensis*, *C. lasiophylla* [20], *C. sylvestris* var. *lingua* [25], and *C. ulmifolia* [20]. Additionally, the Global Natural Products Social Molecular Networking (GNPS) platform offers a way to organize the metabolic profiles of plant and microorganism extracts. GNPS collects similar spectra obtained from high‐resolution mass spectrometry tandem mass spectrometry (HRMS/MS) of compounds in complex mixtures. It constructs molecular networks based on similar MS/MS spectra, forming spectral families or molecular families due to shared fragmentation patterns. This process also suggests analog compounds and biosynthetic pathways, supporting phytochemical studies [26, 27], including the investigation of cytotoxic compounds from plant metabolomes [28].

This work describes the liquid chromatography equipped with diode‐array‐detector (LC‐DAD) guided isolation of flavolignans from *C. arborea* leaves, including the HRMS/MS dereplication of a polar extract fraction using classical molecular networking (CMN) as a reliable computational tool for annotations in the chemical discovery of plant secondary metabolites. Additionally, this work demonstrates the antiproliferative potential of tricin and flavolignan derivatives, such as reducing metabolic activity, limiting colony growth, and inducing cell death.

## 2 Materials and Methods

### 2.1 Plant Materials

Leaves of *C. arborea* (Rich.) Urb. were collected at a specific farm in Alfenas, MG, Brazil, in February 2013 (coordinates 21°21.747′ S 45°50.417′ W) and were named “Paraíso” (**P**). Two additional samples were collected in June 2016 in Alfenas, MG, Brazil, from different farms, and labeled “Gaspar Lopes” (**G**) (21°22′53.8″ S 045°55′46.4″ W) and “Matão” (**M**) (21°30′09.7″ S 045°53′13.5″ W). The plants were identified by taxonomist Marcelo Pólo. They were deposited in the herbarium of the University of Alfenas (UNIFAL‐MG), with the code “1388” for the 2013 collection (Paraíso). The collections from 2016 were stored in the SPF herbarium at São Paulo University, under the codes “Elias, J.P.C. 01” and “Elias, J.P.C. 02,” for Matão and Gaspar Lopes, respectively.

### 2.2 General Experimental Procedures

^1^H and ^13^C‐NMR spectra (including uni‐ and bi‐dimensional) were recorded at 300.13 MHz (^1^H) and 75.77 MHz (^13^C) using an Ultrashield 300 Advance III spectrometer (Bruker‐Biospin, Germany). The instrument was equipped with a 5‐mm trinuclear, inverse detection probe with a z‐gradient (TXI, 5.0 mm). The temperature was maintained at 25°C with a BCU 0.5 I accessory. DMSO‐d_6_ (Sigma‐Aldrich) served as the solvent and internal standard. HRMS/MS data were acquired on a Shimadzu Nexera X2 liquid chromatograph system (Shimadzu, Japan) equipped with an SPD‐M20A Prominence Diode Array detector, coupled to a quadrupole coupled to time‐of‐flight mass analyzer (QTOF) mass spectrometer analyzer (MicroTOF‐QII; Bruker Daltonics, USA) operating with electron spray ionization (ESI) in positive ion mode at 18 000 FWHM for mass resolution. For extracts and compounds, ultra‐performance liquid chromatography coupled to high‐resolution mass spectrometry (UPLC–HRMS) analyses employed a C18 Reverse Phase (RP) Kinetex EVO column (2.6 µm, 100 × 2.1 mm^2^). All solvents used were AR grade (Merck and J. T. Baker), and formic acid was MS grade (Merck). The dichloromethane phase of the methanolic leaf extract was analyzed on an analytical high‐performance liquid chromatography (HPLC) system with an Agilent 1260 liquid chromatograph equipped with a UV‐DAD detector (Agilent), using a Zorbax Eclipse Plus RP column (3.5 µm, 150 × 4.6 mm^2^). Compound purification was performed using a semi‐preparative HPLC system on an Agilent 1260 liquid chromatograph with an automatic sample collector (Agilent), equipped with a UV‐DAD detector (Agilent) and a Zorbax Eclipse Plus column (3.5 µm, 150 × 4.6 mm^2^). All solvents involved in analytical and preparative chromatography were AR grade.

### 2.3 Crude Extract Preparation

The leaves were dried for 48 h in an oven at 40°C. The dry leaves were then ground into powder and extracted with methanol (1 L × 5) at room temperature. After filtration and concentration in a rotary evaporator, the methanolic crude extract of the leaves was obtained. The extract was resuspended in methanol:water (2:1, v/v), and liquid–liquid partitioning was performed with solvents of increasing polarity, producing four fractions: *n*‐hexane, dichloromethane, ethyl acetate, and *n*‐butanol. Each fraction was concentrated using a rotary evaporator at 40°C. From 40 g of **P**, approximately 2.1 g of the dichloromethane phase was obtained, labeled **P_D_**, and from 10 g of **G** and **M**, 0.41 and 0.40 g of the dichloromethane phase were obtained, labeled **G_D_** and **M_D_**, respectively. The dichloromethane fractions **P_D_**, **G_D_**, and **M_D_** were combined (**CarD**) for phytochemical analysis, focusing on purifying flavolignan derivatives. The yield of the dichloromethane fractions was around 4%–5%, the lowest among the extracts, but these fractions were rich in target compounds.

### 2.4 UPLC–HRMS Data Acquisition

A sample of **CarD** was dissolved in methanol, producing 10 mg in 1 mL. The solution was centrifuged at 10 000 rpm for 15 min. The supernatant (100 µL) was transferred to a vial and diluted with 900 µL of a methanol:water (1:1, v/v) mixture to reach a final concentration of 1 mg/mL. It was then analyzed by UPLC–HRMS with a 3 µL injection into the LC system using RP column at 50°C in the column chamber. Separation was achieved with mobile phases (A) water with 0.2% formic acid and (B) acetonitrile (ACN), flowing at 300 µL/min. The gradient method was as follows: 0–1 min, 5% B; 1–4 min, 5%–20% B; 4–8 min, 20% B; 8–12 min, 20%–60% B; 12–14 min, 60%–98% B; and 14–15 min, 98% B. HRMS/MS data were acquired in positive mode ESI over a mass range of *m/z* 75–1200. The ionization parameters were set as follows: capillary tune voltage at 4500 V, end plate offset at 500 V; nitrogen dry gas flow at 8.0 mL/min; pressure at 4.0 bar; and temperature at 200°C. Auto‐MS/MS selected three precursor ions, and the CID ramp energy was based on literature [29], with modifications.

### 2.5 HRMS/MS Data Processing and Molecular Networking Workflow

All HRMS/MS data obtained from the analysis of **CarD** acquired in Bruker microTOF‐QII‐ESI were converted to the “.mzML” extension using the free software MSConvert (Proteowizard) [30, 31] and conferred in TOOPView (OpenMS) [32]. The “.mzML” data were then uploaded to the Mass Spectrometry Interactive Virtual Environment (MassIVE) Web server using WinSCP to create the molecular networking using the GNPS platform with the dataset (MSV000089469, doi:10.25345/C59P2W96N), as well as to perform the dereplication for database matches [26, 29]. To create the molecular networking, the acquired data were treated in the GNPS Data Analysis platform, removing fragments of ±17 Da of precursor *m/z*. HRMS/MS spectra were filtered, choosing only the six top fragments in the ±50 Da window in all ranges of spectra. The basic options for mass tolerance ions were set to 0.02 Da for precursor and QTOF fragment ions and 0.02 Da. A network was then created using the MS‐cluster algorithm [33], according to a cosine score above 0.7, more than two matched peaks, and a minimum cluster size of one spectrum. Further, edges between two nodes were kept in the network if and only if each of the nodes appeared in the other's respective top 10 most similar nodes. The maximum size of a molecular family was set to 100, and the lowest scoring edges were removed from molecular families until the molecular family size was below this threshold. The spectra in the network were then searched against GNPS’ spectral libraries. The library spectra were filtered in the same manner as the input data. All matches kept between network spectra and library spectra were required to have a score above 0.6 and at least three matched peaks. The software Cytoscape was used to visualize and edit the entire molecular networking [34], as well as on the GNPS website (https://gnps.ucsd.edu/ProteoSAFe/static/gnps‐splash.jsp).

### 2.6 Compound Purification

Tricin (**1**) was obtained from previous work using **P_D_** fraction [24] and analyzed by UPLC–DAD–HRMS to guide the purification of tricin derivatives. Flavolignan derivatives were identified by DAD–HRMS/MS spectra in the **P_D_**, **G_D_**, and **M_D_** and then collected into **CarD** for chromatographic purification. The **CarD** was subsequently fractionated by CC using Sephadex LH‐20 as the stationary phase and methanol as the mobile phase. Fractions with higher concentrations of targeted peaks in the flavonoid UV pattern were monitored using an HPLC–DAD system to prepare for the isolation of flavolignan derivatives **2** and **3**. This was performed in a semi‐preparative HPLC–DAD system with mobile phases (A) H_2_O (0.1% acetic acid) and (B) ACN, flowing at 1.0 mL/min. The gradient method was as follows: 0–5 min, 10%–25% B; 5–10 min, 25%–31% B; 10–20 min, 31%–37.5% B; and 20–26 min, 37.5%–100% B. Under these conditions, three bands/peaks were collected at 356 nm detection.

### 2.7 Structural Identification

**Tricin (1)—**DMSO‐d_6_ **^1^H‐NMR 300 MHz, *δ*_H_**: 6.87 (*s*, 1H, H‐3), 6.08 (*d*, 1.5 Hz, 1H, H‐6), 6.41 (*d*, 1.5 Hz, 1H, H‐8), 7.29 (*s*, 2H, H‐2′/6′), 3.87 (*s*, 6H, H‐7′). **^13^C‐NMR 75 MHz, *δ*_C_**: 165.0 (C‐2), 102.6 (C‐3), 181.2 (C‐4), 163.2 (C‐5), 99.5 (C‐6), 164.6 (C‐7), 94.6 (C‐8), 157.6 (C‐9), 103.2 (C‐10), 120.0 (C‐1′), 104.6 (C‐2′/6′), 148.3 (C‐3′/5′), 132.2 (C‐4), 56.3 (C‐7′) [35].

**Tricin‐4**″**‐*O*‐(*threo*‐guaiacylglyceryl) ether, salcolin A (2)**—DMSO‐d_6_ **^1^H‐NMR 300 MHz, *δ*_H_**: 7.0 (*s*, 1H, H‐3), 6.18 (*d*, 1.4 Hz, 1H, H‐6), 6.52 (*d*, 1.4 Hz, 1H, H‐8), 7.29 (*s*, 2H, H‐2′/6′), 3.87 (*s*, 6H, H‐7′), 6.93 (*d*, 1.4 Hz, 1H, H‐1″), 6.69 (*d*, 8.5 Hz, 1H, H‐5″), 6.75 (*dd*, 8.5, 1.4 Hz, 1H, H‐6″), 4.79 (*d*, 4.7 Hz, 1H, H‐7″), 4.34 (*dd*, 8.1, 4.7 Hz, 1H, H‐8″), 3.74 (*dd*, 12.0, 8.1 Hz, 1H, H‐9″), 3.51 (*dd*, 12.0, 8.1 Hz, 1H, H‐9″), 3.74 (*s*, 3H, H‐10″). **^13^C‐NMR 75 MHz, *δ*_C_**: 165.0 (C‐2), 103.4 (C‐3), 181.7 (C‐4), 161.4 (C‐5), 99.5 (C‐6), 162.9 (C‐7), 94.6 (C‐8), 157.5 (C‐9), 104.2 (C‐10), 119.4 (C‐1′), 104.7 (C‐2′/6′), 147.0 (C‐3′/5′), 133.2 (C‐4), 56.4 (C‐7′), 133.2 (C‐1″), 110.8 (C‐2″), 147.0 (C‐3″), 145.4 (C‐4″), 114.6 (C‐5″), 119.6 (C‐6″), 72.0 (C‐7″), 86.3 (C‐8″), 60.2 (C‐9″), 55.5 (C‐10″) [36].

**Tricin‐4**″**‐*O*‐(*erythro*‐guaiacylglyceryl) ether, salcolin B (3)—**DMSO‐d_6_ **^1^H‐NMR 300 MHz, *δ*_H_**: 6.87 (*s*, 1H, H‐3), 6.12 (*d*, 1.0 Hz, 1H, H‐6), 6.46 (*d*, 1.0 Hz, 1H, H‐8), 7.29 (*s*, 2H, H‐2′/6′), 3.85 (*s*, 6H, H‐7′), 6.98 (*d*, 1.6 Hz, 1H, H‐2″), 6.69 (*d*, 8.0 Hz, 1H, H‐5″), 6.79 (*dd*, 8.1, 1.6 Hz, 1H, H‐6″), 4.84 (*d*, 4.3 Hz, 1H, H‐7″), 4.24 (*dd*, 9.5, 4.4 Hz, 1H, H‐8″), 3.80 (*dd*, 12.0, 9.5 Hz, 1H, H‐9″), 3.64 (*dd*, 12.0, 4.4 Hz, 1H, H‐9″), 3.85 (*s*, 3H, H‐10″). **^13^C‐NMR 75 MHz, *δ*_C_**: 165.0 (C‐2), 103.4 (C‐3), 181.7 (C‐4), 161.4 (C‐5), 99.5 (C‐6), 162.9 (C‐7), 94.6 (C‐8), 157.5 (C‐9), 104.2 (C‐10), 119.4 (C‐1′), 104.7 (C‐2′/6′), 147.0 (C‐3′/5′), 133.2 (C‐4), 56.4 (C‐7′), 133.5 (C‐1″), 111.7 (C‐2″), 148.7 (C‐3″), 147.2 (C‐4″), 115.9 (C‐5″), 120.9 (C‐6″), 74.4 (C‐7″), 88.8 (C‐8″), 62.0 (C‐9″), 56.4 (C‐10″) [36].

### 2.8 Cell Culture

Two thyroid cancer cell lines were used to assess the antitumor activity of the compounds. HTH83 is derived from ATC, whereas TPC‐1 originates from PTC, each harboring different gene mutations. Both were grown in RPMI medium supplemented with 10% fetal bovine serum at 37°C with 5% CO_2_ [37]. The medium was changed every 48 h, and when necessary, cells were detached using Trypsin‐EDTA (0.25%) (Gibco) when needed. Prof. Edna Kimura kindly donated the cell lines from the University of São Paulo.

### 2.9 IC_50_ Determination

The concentration that reduces cell viability to 50% (IC_50_) was determined after 72 h of treatment using PrestoBlue Cell Viability Reagent (Thermo Fisher Scientific), following the manufacturer's instructions. Briefly, cell lines were seeded into 96‐well plates at an initial density of 3000 cells and incubated at 37°C with 5% CO_2_ for 24 h. Cells were then treated with compounds **1**–**3** in a serial dilution starting at 250 µM. After 72 h of treatment, 10% of PrestoBlue reagent was added to each well. Plates were incubated for 1 h and 30 min at 37°C with 5% CO_2_, protected from light. Fluorescence (540 nm excitation, 590 nm emission) was measured using a microplate reader M3 (molecular devices). The relative luminescence units of treated cells were normalized to the fluorescence of vehicle‐treated control cells (DMSO) and expressed as a percentage of viable cells. IC_50_ was determined from a nonlinear regression analysis of the dose–response curve using Prism 5 (GraphPad software).

### 2.10 Clonogenic Assay

To evaluate colony formation after treatment, 250 cells were seeded in a 6‐well plate and incubated for 24 h at 37°C with 5% CO_2_. Next, the cells were treated with the IC_50_ of compounds **1**–**3** for 72 h, then cultured for more than 10–15 days, with media changed twice a week without treatment. Each well was then washed with 1 mL of PBS, fixed with a methanol (33%) and acetic acid (33%) solution for 10 min at 4°C, and stained with 0.5 mL of 1% crystal violet for another 10 min at room temperature. Excess crystal violet was washed off with distilled water, and plates were allowed to dry at room temperature. The number of colonies was counted manually, and the treated groups were normalized against the number of colonies in the DMSO‐treated group.

### 2.11 Annexin‐V Assay

Annexin‐V‐FITC/Propidium Iodide (PI) kit (Thermo Fisher Scientific) was used to investigate which cell death mechanism could be activated after treatments. Briefly, 2 × 10^5^ cells were seeded into a 6‐well plate and incubated for 24 h. Cells were treated with the IC_50_ of compounds **1**–**3** for 48 h. Cells were detached with trypsin‐EDTA (0.25%) (Gibco), and 1 × 10^5^ cells were incubated for 30 min, in the dark, at 37°C with Annexin‐V‐FITC (0.2 mg/mL) and PI (0.05 µg). Then, samples were analyzed in a flow cytometer (detection at 488 and 617 nm wavelengths) (Becton Dickinson, FACSCalibur).

## 3 Results

### 3.1 Phytochemical Characterization

The dichloromethane phase obtained from the liquid–liquid partition of methanolic extracts (**CarD**) was analyzed by UPLC–HRMS to determine the chemical profile. The DAD chromatogram of the dichloromethane fraction showed multiple UV spectra indicating the flavone backbone chromophore (*λ*_max_ 270, 330). The mass spectra confirmed the presence of flavone tricin and its derivatives, based on HRMS/MS fragments of positive ion *m/z* 331. This flavonoid was previously identified in the dichloromethane phase of *C. arborea* leaves by our group [24]. Two tricin derivative ions were detected (*m/z* 527) in the **CarD** sample using HR‐ESI positive mode, as well as in negative mode (*m/z* 525), suggesting the structures of two tricin‐4′‐*O*‐guaiacylglyceryl ethers according to the literature [36]. Accordingly, the **CarD** was subjected to semi‐preparative purification via HPLC–DAD to isolate the flavolignans for cytotoxic assays.

### 3.2 Dereplication

**FIGURE 1:**
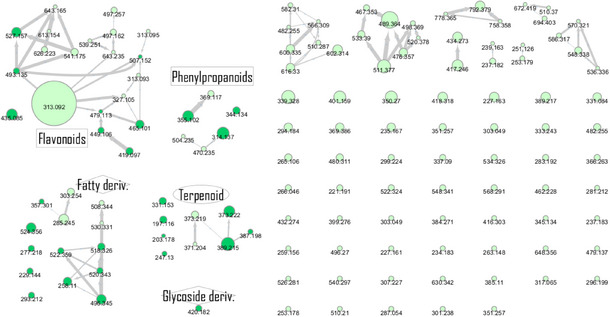
Chemical profile of the dichloromethane fraction of *Casearia arborea* leaves presented as a classical molecular network. The green nodes indicate mass spectra detected in the **CarD** sample. Dark green nodes represent annotated cluster spectra. Nodes with MS2 cosine similarity above 0.7 were connected. Spectra detected in the blank sample were removed.

The chemical profile of the **CarD** was organized using CMN, and dereplication was performed on the basis of ESI‐(+)‐HRMS/MS analysis and GNPS. A total of 39 annotations were organized, including 29 spectral annotations from the GNPS dereplication workflow and 10 spectral annotations based on the CMN spectral organization, through MS/MS analysis of correlated cluster spectra (Figure 1, Table 1).

**TABLE 1:** Chemical profile of dichloromethane fraction from leaves of *Casearia arborea*.

Code	RT (s)	Annotation	Parent mass (*m/z*) Ion formula Adduct	Cosine (GNPS)	Error (ppm)	HRMS/MS CID
A1	52.8	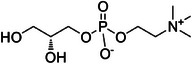 Choline alfoscerate	258.110 C_8_H_21_NO_6_P^+^ [M + H]^+^	0.93	0	104.11 184.07 125.00
A2	204.8	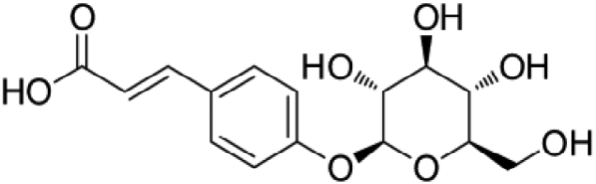 4‐*O*‐β‐d‐glucosyl‐4‐coumaric acid	344.134 C_15_H_22_NO_8_^+^ [M + NH_4_]^+^	0.89	0	147.04 165.05 85.03 127.04
A3	224.3	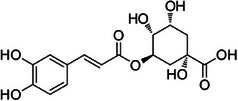 Chlorogenic acid	355.102 C_16_H_19_O_9_^+^ [M + H]^+^	0.98	0	163.04 181.05
A4	239.5	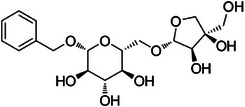 Icariside F2	420.182 C_18_H_30_NO_10_^+^ [M + NH_4_]^+^	0.81	9.5	115.04 133.05 91.05
A5	257.7	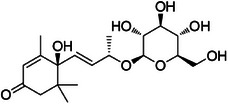 Corchoionoside C	387.198 C_19_H_33_O_7_^+^ [M + H]^+^	0.66	7.7	209.15 (207) 149.09 191.14 123.09 (125) 135.11 95.08
A6	285.0	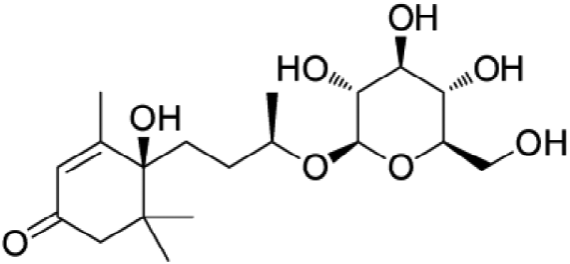 Icariside B5	389.215 C_19_H_33_O_8_^+^ [M + H]^+^	0.81	2.6	209.15 149.10 (151) 191.14 125.09 227.16
A7	309.0	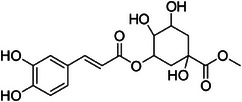 Chlorogenic acid methyl ester	369.117 C_17_H_21_O_9_^+^ [M + H]^+^	—	2 Calcd. 369.1186	163.04 181.05
A8	330.1	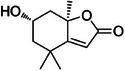 Loliolide	197.116 C_11_H_17_O_3_^+^ [M + H]^+^	0.78	5.1	133.10 (135) 107.08 161.09
A9	338.3	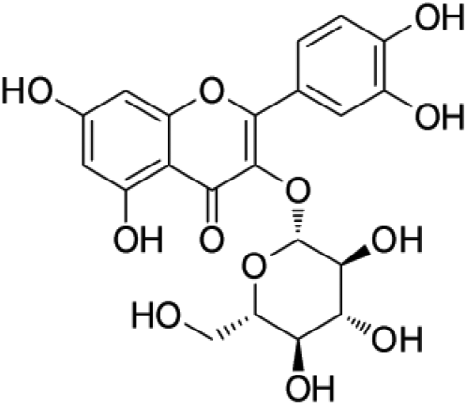 Isoquercetin	465.101 C_21_H_21_O_12_^+^ [M + H]^+^	0.86	2.4	303.05 153.13
A10	350.6	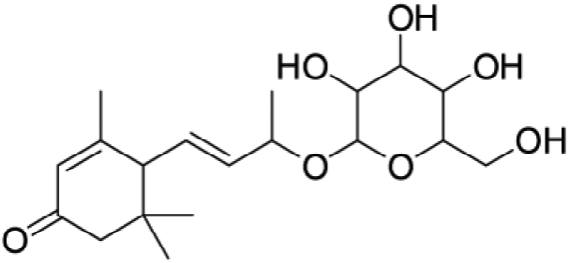 (6*R*,9*S*)‐3‐*oxo*‐*α*‐ionol‐β‐d‐glucoside (from PubChem)	373.219 C_19_H_31_O_7_^+^ [M + H]^+^	—	35.0 Calcd. 371.2064	133.10 (135, 137) 193.16 (191) 211.17 (207, 209) 151.04 109.10
A11	363.1	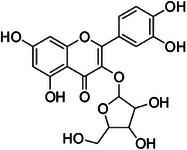 Quercetin‐3‐*O*‐pentoside	435.085 C_20_H_19_O_11_^+^ [M + H]^+^	0.92	0	303.05 237.18
A12	365.0	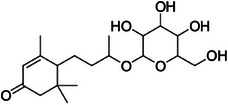 Byzantionoside B isomer	373.219 C_19_H_33_O_7_^+^ [M + H]^+^	—	8.0 Calcd. 373.2221	133.10 (135, 137) 191.14 209.15 151.11 109.10
A13	377.2	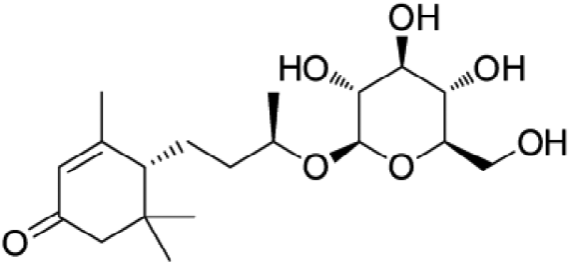 Byzantionoside B or Blumenol‐C‐glucoside	373.222 C_19_H_33_O_7_^+^ [M + H]^+^	0.91	0	211.17 193.16 135.12 109.10 175.15 119.09
A14	408.2	Benzoic acid derivative	313.092 [M + H]^+^	—	—	151.04
A15	419.7	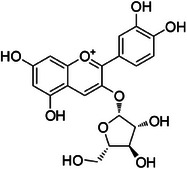 Cyanidin‐3‐*O*‐α‐arabinoside	419.097 C_20_H_19_O_10_^+^ M^+^	0.90	0	287.05
A16	463.0	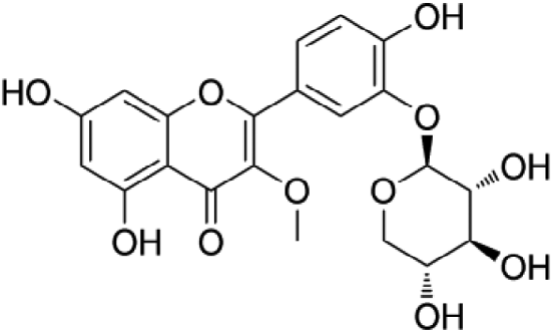 Quercetin‐3‐*O*‐methoxy 3′‐*O*‐β‐d‐xylopyranose	449.106 C_21_H_21_O_11_^+^ [M + H]^+^	0.87	4.5	317.06
A17	468.0	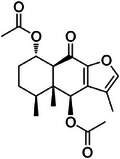 1,6‐di‐Acetyl‐furannoeremophilone	331.153 C_19_H_23_O_5_^+^ [M + H − H_2_O]^+^	0.62	0	151.07 (149) 137.06 285.11 (287) 255.10 227.10 189.09
A18	479.2	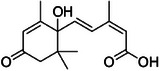 Abscisic acid	247.130 C_15_H_19_O_3_^+^ [M + H − H_2_O]^+^	0.60	12.1	187.11 201.13 121.07 (119)
A19	506.7	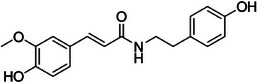 Feruloyltyramine	314.137 C_18_H_20_NO_4_^+^ [M + H]^+^	0.96	6.4	177.05 121.07 145.03 151.04
A20	550.1	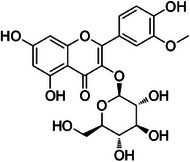 Isorhamnetin 3‐*O*‐glucoside	479.113 C_22_H_23_O_12_^+^ [M + H]^+^	0.89	10.4	317.06
A21	573.5	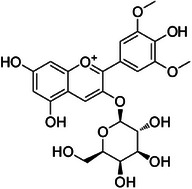 Malvidin 3‐*O*‐galactoside	493.135 C_23_H_25_O_12_^+^ M^+^	0.84	4.1	331.08 151.04
A22	588.1	Phenylpropanoid derivative Similar fragments of feruloyltyramine compound	626.223 [M + H]^+^	—	—	314.14 177.05 151.04
A23	620.2	Feruloyltyramine derivative	504.235 [M + H]^+^	—	—	177.05 151.04 251.12 328.19
A24	622.8	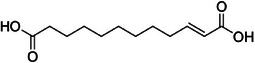 Traumatic acid	229.144 C_12_H_21_O_4_^+^ [M + H]^+^	0.72	2.1	147.12 165.13 123.12 175.11
A25	646.4	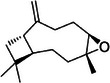 β‐Caryophyllene oxide	203.178 C_15_H_23_^+^ [M + H − H_2_O]^+^	0.73	9.8	119.09 95.09 105.07 (107) 81.07
A26	655.6	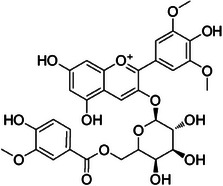 Malvidin derivative	643.165 C_31_H_31_O_15_^+^ [M + H]^+^	—	1.0 Calcd. 643.1657	331.08 151.04
A27	656.9	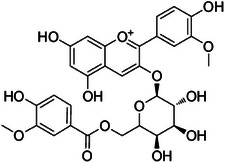 Peonidin derivative	613.154 C_30_H_29_O_14_^+^ [M + H]^+^	—	2 Calcd. 613.1552	301.07 151.04
A28	676.2	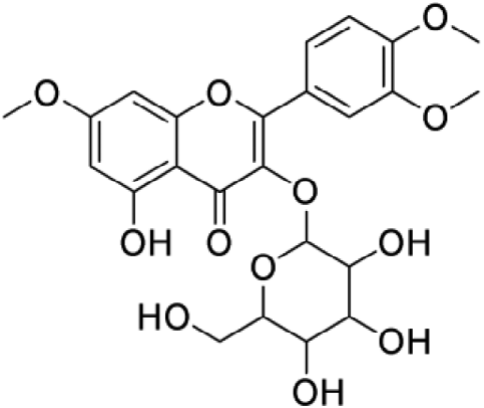 Quercetin‐3‐*O*‐hexoside‐7,3′,4′‐trimethyl ether	507.152 C_24_H_27_O_12_^+^ [M + H]^+^	0.76	5.9	345.10 151.04
A29	676.4	Feruloyltyramine derivative (+C_6_H_12_N_4_O)	470.235 C_24_H_32_N_5_O_5_^+^ [M + H]^+^	—	11.3 Calcd. 470.2398	177.05 294.19
A30	716.0	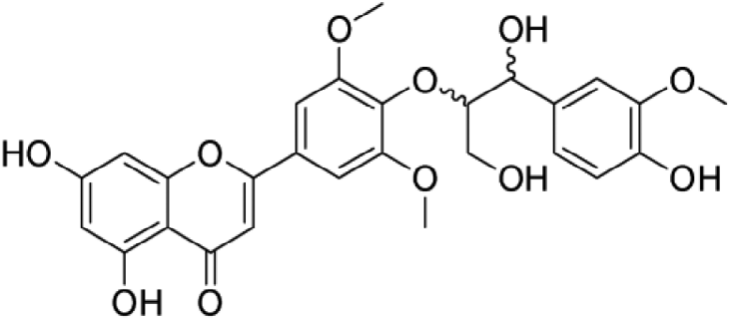 Salcolin or tritin‐4′‐*O*‐guaiacyglyceryl ether	527.157 C_27_H_27_O_11_^+^ [M + H]^+^	0.83	1.8	331.08
A31	747.7	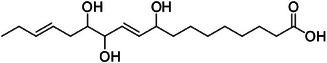 9,12,13‐Trihydroxy‐10,15‐octadecadienoic acid (9,12,13‐TriHODE)	293.212 C_18_H_29_O_3_^+^ [M + H − 2H_2_O]^+^	0.61	3.4	107.08 147.02 81.06 151.04
A32	761.5	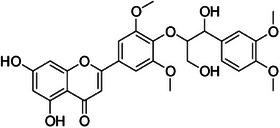 Tricin‐4′‐*O*‐veratrylglyceryl ether	541.175 C_27_H_27_O_11_^+^ [M + H]^+^	—	37.0 Calcd. 541.1548	331.08
A33	846.1	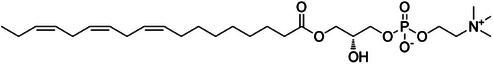 LPC (18:3/0:0) or γ‐Linoleate phosphotidylcholine	518.326 C_26_H_49_NO_7_P^+^ [M + H]^+^	0.86	2.6	184.07 104.11
A34	871.1	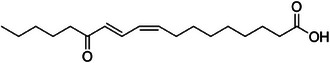 13‐Keto‐9*Z*,11*E*‐octadecadienoic	277.218 C_18_H_29_O_2_^+^ [M + H − H_2_O]^+^	0.68	10.8	149.07 135.12 121.10 93.07 (95)
A35	875.1	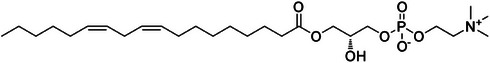 LPC (18:2/0:0)	520.343 C_18_H_29_O_2_^+^ [M + H]^+^	0.89	5.2	184.07 104.11
A36	896.5	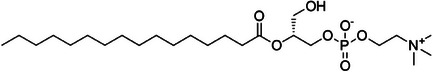 PC (0:0/16:0)	496.345 C_24_H_51_NO_7_P^+^ [M + H]^+^	0.89	9.5	184.07 104.11 151.04
A37	897.5	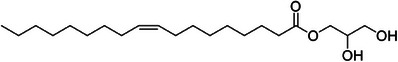 Monoolein	357.301 C_21_H_39_O_4_^+^ [M + H]^+^	0.71	2.8	95.09 (97) 109.10 135.12 83.08
A38	907.5	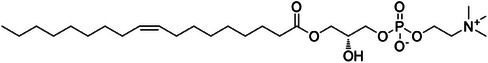 1‐Oleoyl‐sn‐glycero‐3‐phosphocholine	522.359 C_26_H_51_NO_7_P^+^ [M + H]^+^	0.78	11.4	184.07 104.11 283.26
A39	950.1	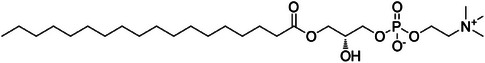 1‐Stearoyl‐sn‐glycero‐3‐phosphocholine	524.356 C_26_H_53_NO_7_P^+^ [M + H]^+^	0.60	24.8	313.28 184.07 239.24 104.11

*Note*: Spectral annotations of Global Natural Products Social Molecular Networking (GNPS) library hits based on UPLC‐ESI‐(+)‐HRMS‐MS^2^ results of CarD sample analysis.

The dichloromethane phase **CarD** showed a polar profile with shikimate derivative compounds, as indicated by spectral annotations of two benzoic compounds (**A4** and **A14**), phenylpropanoids (**A2**, **A3**, **A7**, **A19**, **A22**, **A23**, and **A29**) (Supporting Information S1), and flavonoids (**A9**, **A11**, **A15**, **A16**, **A20**, **A21**, **A26**, **A27**, and **A28**) or flavolignans (**A30** and **A32**) (Supporting Information S2). Apolar compounds, including terpene derivatives such as one carotenoid (**A8**), one eremophilane (**A17**), one sesquiterpenoid (**A25**), and six megastimane derivatives (**A5**, **A6**, **A10**, **A12**, **A13**, and **A18**), were also identified (Supporting Information S3). Additionally, the apolar compounds in the **CarD** fraction included fatty acid derivatives, annotated as **A1**, **A24**, **A31**, and **A33**–**A39** (Supporting Information S4).

### 3.3 Structural Identification of Compounds **1–3** by NMR and HRMS/MS

Compound **1** was identified as tricin by HR‐ESI‐(+) with *m/z* 331.0800 and NMR data. Additionally, the structure of tricin in flavolignans 2 (CarD‐9/2) and 3 (CarD‐9/3) was confirmed by ^1^H and ^13^C‐NMR according to the literature [36]. Compounds **2** and **3** showed *m/z* 527.1572 and 527.1581, respectively, consistent with the [M + H]^+^ adduct and the molecular formula C_27_H_26_O_11_ (exact mass 527.1553, Supporting Information S5). The negative mode further verified the tricin derivative, with [M–H]^−^ matching *m/z* 525.1439 (same molecular formula, C_27_H_26_O_11_). HR‐ESI‐(−)‐MS/MS analysis revealed the guaiacyl fragment, based on the proposed fragmentation mechanism (Supporting Information S6). The fragment at *m/z* 329.0653 in the negative mode spectrum corresponds to the tricin anion, indicating a guaiacyl structure (*m* 196), confirmed by the negatively charged fragment at *m/z* 195.0657.

**FIGURE 2:**
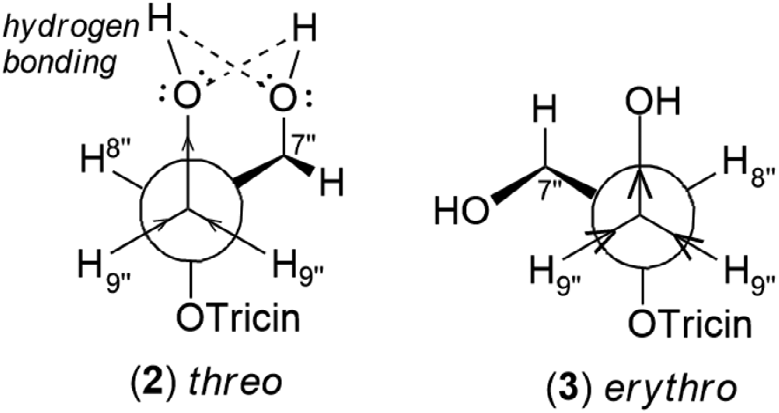
Using Newman projection to explain the H‐9″ deshielded proton shift in ^1^H‐NMR in the *erythro* diastereomer **(3)**. The *threo* **(2)** exhibits less inductive effect because of hydrogen bonding, which is absent in *erythro* (**3**).

To confirm the flavolignan structures, compounds **2** and **3** were analyzed using ^1^H and ^13^C‐NMR spectra, showing tricin chemical shifts along with signals assigned to the coniferyl acid derivative through HSQC spectra. The aromatic region includes *δ*_H‐2″_ 6.9/*δ*_C‐2″_ 110.8, *δ*_H‐5″_ 6.7/*δ*_C‐5″_ 114.6, and *δ*_H‐6″_ 6.75/*δ*_C‐6″_ 119.2. Carbinolic carbons appear at *δ*_H‐7″_ 4.8/*δ*_C‐7″_ 72.0, *δ*_H‐8″_ 4.3/*δ*_C‐8″_ 86.3, and *δ*_H‐9″_ 3.5 and 3.7/*δ*_C‐9″_ 60, along with one aromatic methoxyl group at *δ*_H‐10″_ 3.7/*δ*_C‐10″_ 55.3. The 4′‐*O*‐linkage was determined via NOESY spectra (Supporting Information S7), where proton couplings between methoxyl hydrogen of tricin (H‐7′) and guaiacyl (H‐7″, H‐8″, H‐9″) were observed at *δ*_H‐7′_ 4.0/*δ*_H‐7″_ 5.0, *δ*_H‐7′_ 4.0/*δ*_H‐8″_ 3.9, and *δ*_H‐7′_ 4.0/*δ*_H‐9″_ 3.9. Compound **3** (*erythro*) displayed a slightly deshielded H‐9″ compared to **2** (*threo*), according to the literature [36]. Furthermore, the Newman projection of both diastereoisomers helps visualize the relative positions of hydroxyl groups in each configuration. In compound **2** (*threo*), the hydroxyl groups are in the same spatial orientation, enabling hydrogen bonding that minimizes the inductive effect on H‐9″ and H‐7″. Conversely, compound **3** (*erythro*) has the 7″‐OH near H‐9″, but no hydrogen bond occurs with 9″‐OH. The higher inductive effect of 9″‐OH, due to the absence of hydrogen bonding with 7″‐OH, results in deshielding of H‐9″ (Figure 2).

### 3.4 Cytotoxic Potential for Compounds **1–3**

**FIGURE 3:**
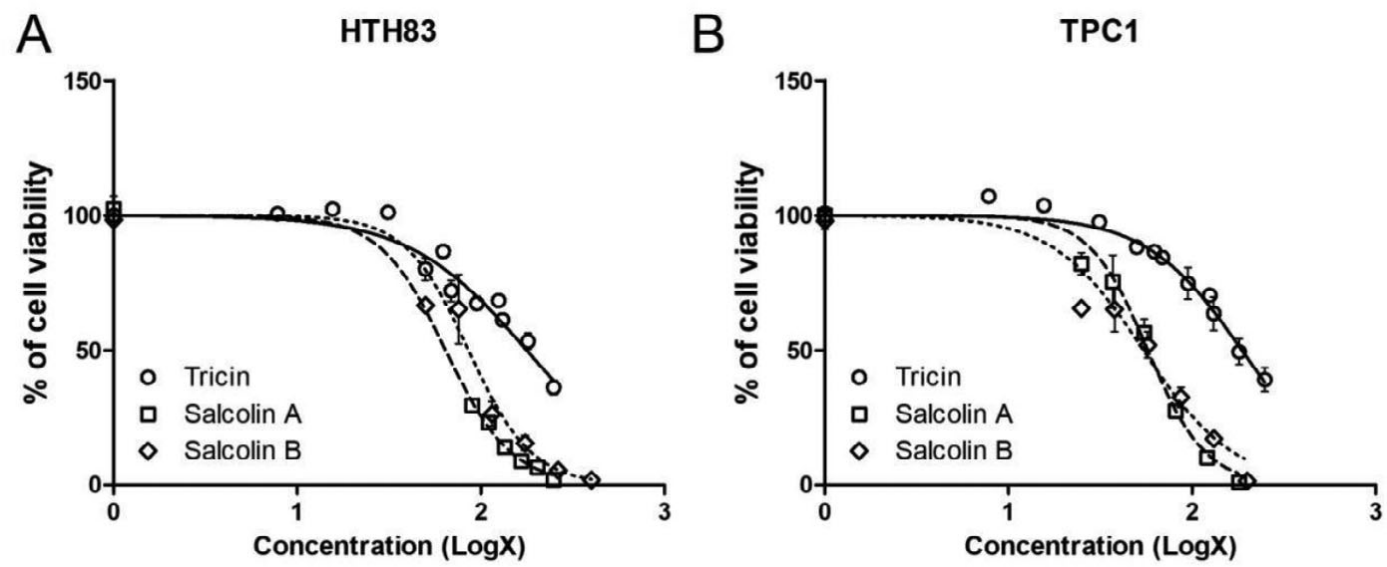
Tricin, salcolin A, and salcolin B reduced cell viability in HTH83 (A) and TPC1 (B) after 72 h treatment (mean + SD of at least three independent experiments).

Tricin (**1**), salcolin A (**2**), and salcolin B (**3**) were tested against thyroid cancer cell lines (Table 2) and shown to decrease cell viability (Figure 3). The IC_50_ values for each compound in each cell line are listed in Table 3. Tricin (**1**) had similar IC_50_ values in HTH83 and TPC1 (177.5 and 187.0 µM, respectively); however, lower concentrations of compounds **2** and **3** were needed to reduce cell viability by 50% in both cell lines (66.69 and 84.98 µM for HTH83, respectively; 56.12 and 56.5 µM for TPC1, respectively).

**TABLE 2:** Cell lines used in the study and the harboring mutations.

Cell line	Type	Mutations
HTH83	ATC	AR p.G456_G457insG; HRASQ61R; TERT c.228C>T; TP53 p.P154Afs*28
TPC1	PTC	RET/PTC1; CDKN2A p.A68fs; STAG2Q1089X; TERT c.228C>T

Abbreviations: ATC, anaplastic thyroid cancer; PTC, papillary thyroid cancer.

**TABLE 3:** IC_50_ (µM) determined for HTH83 (anaplastic thyroid carcinoma cells—ATC) and TPC‐1 (papillary cancer cells—PTC).

Compounds	HTH‐83	TPC‐1
Tricin (**1**)	177.5	187.0
Salcolin A (**2**)	66.69	56.12
Salcolin B (**3**)	84.98	56.5

*Note*: The IC_50_ value was calculated using non‐linear regression in GraphPad Prism 5.0.

**FIGURE 4:**
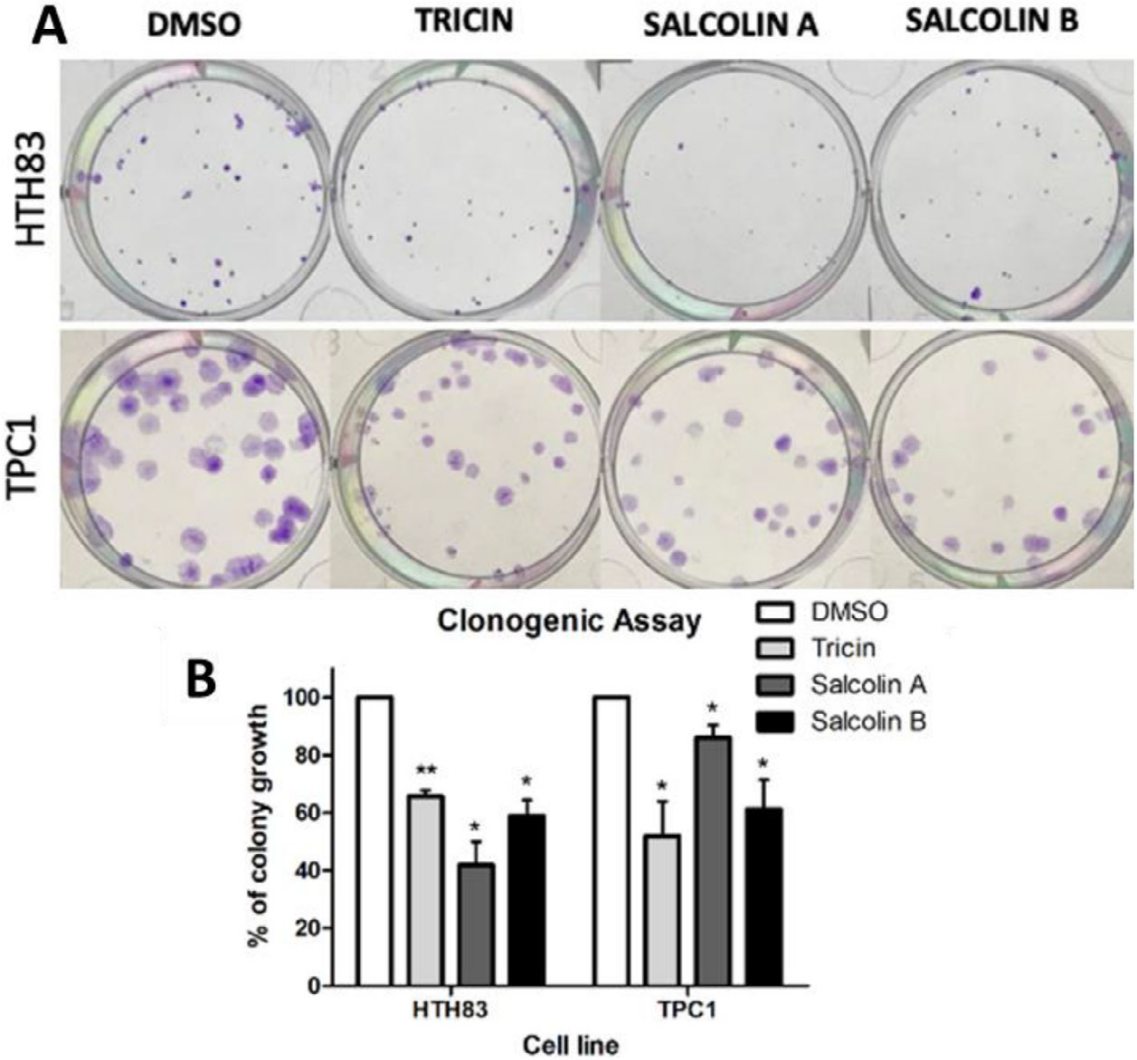
Tricin, salcolin A, and salcolin B reduced colony growth in HTH83 and TPC1 cell lines after 72 h of treatment (A). Graphs were plotted as mean + SD of at least two and three independent experiments, respectively. *p < 0.05; **p < 0.01 (B).

To confirm the reduction in cell viability, clonogenic assays were performed using the IC_50_ concentration of each compound to treat both cell lines (Figure 4). Compound **1** decreased colony growth by 34.58% in HTH83 and 48.04% in TPC1 (*p* < 0.01 and *p* < 0.05). Compound **2** reduced colony growth by 58.05% in HTH83 and 13.97% in TPC1 (*p *< 0.05), whereas Compound **3** decreased it by 41.09% in HTH83 and 38.68% in TPC1 (*p* < 0.05). Similar results were reported for tricin in other cancer histology samples. A reduction in proliferation, colony growth, and cell cycle arrest was observed in a breast cancer cell line [38]. In a colon cancer cell line, treatment with tricin lowered the number of colonies [39].

**FIGURE 5:**
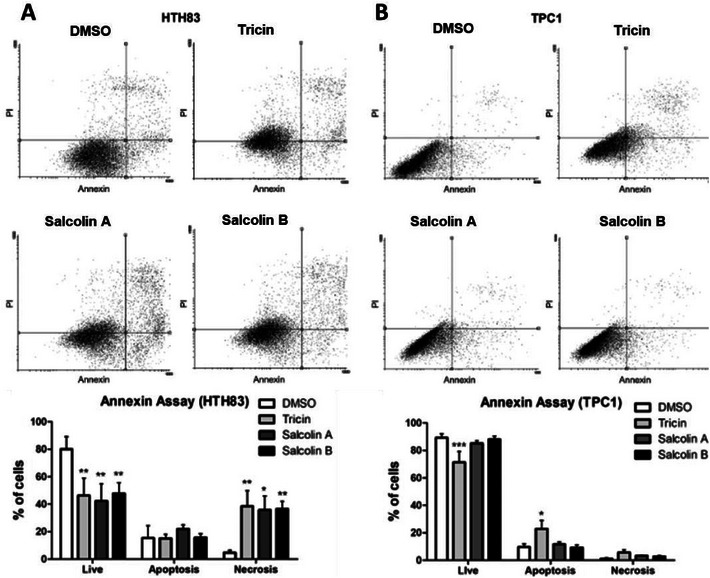
Tricin, salcolin A, and salcolin B treatments reduced the percentage of live cells and increased necrotic cells in HTH83 (A), whereas tricin treatment decreased live cells and increased cells in apoptosis in TPC1 (B). The graphs show cell distribution after DMSO, tricin, salcolin A, and salcolin B treatment (mean + SD of at least three experiments). Live: cells annexin−/PI−; apoptosis: cells annexin+/PI− and annexin+/PI+; necrosis: cells annexin/PI+. *p < 0.05; **p < 0.01; ***p < 0.001.

To better understand that cell death mechanism might be activated after flavone treatments, Annexin‐V assays were conducted. Treatment with compounds **1**–**3** reduced the percentage of live cells (cells annexin−/PI−) by 33.73%, 37.67%, and 32.23%, respectively, in HTH83 (*p* < 0.01) (Figure 5A), whereas the percentage of necrotic cells (cells annexin−/PI+) increased to 31.83%, 31.09%, and 33.89%, respectively (*p* < 0.01, *p* < 0.05, and *p* < 0.01) (Figure 5A). Compound **1** was the only compound that reduced the percentage of live cells in TPC1 (17.81%, *p* < 0.001), and it increased the percentage of apoptotic cells (cells annexin+/PI− and annexin+/PI+) by 13.29% (*p* < 0.05) (Figure 5B).

## 4 Discussion

The chemical diversity of the dichloromethane fraction from leaves of *C. arborea* is similar to *C. sylvestris* var. *lingua* [25], presenting flavonoid‐3‐*O*‐glycosides, phenylpropanoids, and flavolignans. Our group has also identified flavone‐3‐*O*‐glycosides in the ethyl acetate fraction from *C. arborea* [23]. Here, we report the chemical profile of the dichloromethane phase from leaves of *C. arborea* methanolic extracts, including the presence of tricin and rare flavolignans presenting 4′‐*O*‐8″‐guaiacylglyceryl derivatives. The free occurrence of tricin may be linked to enzymatic hydrolysis of tricin derivatives during extraction [40], as seen in the extraction of tricin from bamboo, palms, sugarcane, grains, seeds [41], and plant juice [42]. The first natural tricin flavolignan derivative was reported from *Aegilops ovata*, named aegicin (4′‐*O*‐*p*‐coumaroylglyceryl ether), which inhibits plant germination [43]. Two flavolignans, salcolins A and B, were isolated from *Salsola collina*, with tricin detected in large amounts. These compounds were obtained with *threo* and *erythro* configurations of tricin‐4′‐*O*‐guaiacylglyceryl ether [44]. Tricin and its derivatives are common among many monocotyledonous plants, such as Gramineae [45] and Cyperaceae [46, 47]. Tricin has biological activities related to plant protection. Its accumulation boosts wheat seedlings treated with herbicide safener [48], and it has demonstrated antibacterial, antifungal, and insecticidal properties [49]. Tricin and its derivatives also exhibit antioxidant, anti‐plant‐hopper, anti‐weed, and anti‐herbicide effects, act as gene inducers, protect against biotic and abiotic stresses, and show allelopathic activity [50, 51].

Tricin and its derivatives have pharmaceutical potential due to their antioxidant activity, ability to act as anti‐inflammatory agents, inhibit exocytosis from antigen‐stimulated rat leukemia basophils and hepatitis B, serve as chemopreventive agents against intestinal carcinogenesis, and inhibit breast cancer cells, among other applications [50]. They inhibit P‐glycoprotein activity in adriamycin‐resistant human breast cancer cells, delay spontaneous mammary tumor development, and suppress oxidative stress‐induced apoptosis [52]. Tricin exhibits anti‐clonogenic activity in vitro and has been reported for use in treating human‐derived breast (MDA‐MB‐468) and colon carcinoma (SW‐480) [39], including antineoplastic properties in vivo against P388 leukemia [53] and promyelocytic leukemia cells (HL‐60) [54]. It was shown to promote cell cycle arrest in the MDA‐MB‐486 cell line without inducing apoptosis [38]. In glioma cells (C6), tricin inhibited proliferation and invasion [55]. In Lewis lung carcinoma, tricin inhibited proliferation and migration and induced apoptosis [56]. Tricin improved docetaxel efficacy in prostate cancer cells (PCSs) [57].

Our results show that tricin inhibits the growth of thyroid cancer cell lines. Different results were observed in the two studied cell lines. In the ATC cell line, there is an increased necrotic effect, whereas in the PTC cell line, there is evidence of apoptotic cell death. PTC is the most common type of thyroid cancer and generally has a good prognosis [58]. Approximately 70% of PTCs have activation of the MAPK pathway due to BRAF and RAS mutations, as well as RET fusions, which promote cancer progression [59]. TPC1, a PTC cell line, contains the RET/PTC1 rearrangement, which affects several transcription factors involved in cell proliferation, differentiation, and apoptosis [60]. Cancer cells with RET/PTC1 rearrangements rely more on the RAF‐1 pathway for survival and growth than on the BRAF‐ERK pathway, which is typically needed in cells with RAS or BRAF mutations [61]. ATC is rare and the most aggressive form of thyroid cancer (accounting for 1%–2% of cases), with a survival time ranging from 0.5 to 3 years [62]. HTH83 is a cell line derived from ATC that contains the H‐Ras Q61R mutation, which is associated with the MEK‐ERK and PI3K/AKT pathways, both involved in cancer progression [63]. We hypothesize that the different mutation profiles of HTH83 and TPC1 influence their responses to tricin in this study. Tricin successfully reduced the number of live cells and increased apoptotic cells in TPC1, BRAF‐mutated cells. However, it did not induce significant apoptosis in HTH83, instead causing only necrosis in this cell line harboring the H‐RAS mutation. These findings prompt us to consider whether flavolignans induce cell death or simply decrease cell viability in TPC1.

In an in vivo experiment, Cai et al. [64] reported a controlled diet, including tricin, in mice. The consumption of food supplemented with tricin reduced the number of intestinal adenomas and the mechanisms by which tricin inhibits or downregulates the COX‐2 enzyme and decreases PGE_2_ production. The potential of flavonoids for prevention and chemoprevention is well known [65, 66, 67, 68]. Seki et al. [69] suggest tricin as a therapeutic prototype for hepatic fibrogenesis in in vivo assays. The flavone inhibits PDGF‐induced proliferation of HSCs in vitro by blocking PDGF‐R phosphorylation and downstream signaling pathways rather than by competing for PDGF receptor binding. An acylated tricin glucoside demonstrated antioxidant and antiproliferative activities against breast (MCF‐7, NCI‐ADR), prostate (PC3), ovary (OVCAR‐03), non‐small cell lung (NCI460), and colon cancer cell lines (HT‐29). In melanoma (UACC‐62) and kidney cell lines (786‐0), activity was only observed at high concentrations [70]. The compound 5,7,3′,4′,5′‐pentamethoxyflavone (PMF) contains methoxy groups, which appear to enhance metabolic stability, resulting in slower removal compared to tricin [71].

Mohanlal et al. [72] first reported the cytotoxicity of salcolins A and B in vitro against HCT 116 (colon), SKOV3 (ovary), and MCF‐7 (breast) human cancer cell lines. Apoptosis was observed and confirmed by the decreased mitochondrial membrane potential. Another activity of salcolin B was a dose‐dependent inhibition of nitric oxide production in macrophage cell lines without evidence of cytotoxicity [73], along with higher anti‐inflammatory and anti‐allergy activities of tricin derivatives, including salcolins A and B, compared to tricin [74], and neuroprotective effects [75]. Our results show that for ATC, both salcolins A and B induced necrotic cell death at the same levels as tricin but at lower concentrations. In the PTC cell line, salcolins A and B did not significantly induce apoptosis or necrosis, unlike tricin. These findings highlight the influence of cell genotype on the biological activity of the flavolignan derivatives.

Regarding the structural identification of flavolignan tricin derivatives, Yan et al. [76] reported the identification of lycopodone, a rare compound with no activity against A549 and K562 tumor cell lines. Still, tricin showed moderate cytotoxic activity against K562. Yan et al. used HMBC projection to conclude that the 2D‐NMR flavolignan coupling is only visible in ROESY or NOESY experiments [36, 77]. We also used NOESY to observe the correlation of the 4′‐*O*‐linkage, defined in proton couplings between methoxyl hydrogen of the B ring of tricin (H‐7′) and guaiacyl (H‐7″, H‐8″, H‐9″), to confirm the tricin‐4′‐*O*‐8″‐guaiacylglyceryl linkage in the salcolin diastereoisomers, which is different from the lycopodone reported by Yan et al.

**FIGURE 6:**
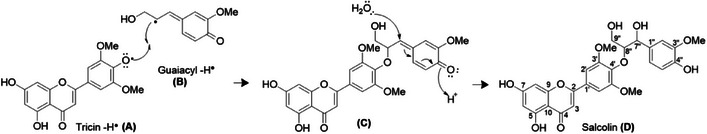
Proposed biogenesis mechanisms for the tricin‐4′‐*O*‐7″‐guaiacylglyceryl linkage. Tricin radical (A) and guaiacyl radical (B) react through radical coupling to form the intermediate 4′‐*O*‐8″‐guaiacylglyceryl linkage. (C) Enzymatic hydrolysis and nucleophilic attack reactions recover the guaiacyl aromatic system. (D) Structure of the flavolignan salcolin.

The tricin‐4′‐*O*‐7″‐guaiacylglyceryl linkage in lycopodone initially appears incorrect because the preferred biogenesis of the tricin‐4′‐*O*‐guaiacylglyceryl linkage occurs at C‐8″ of guaiacol glyceryl (Figure 6), unless the radical is absent at C‐7″, as confirmed by the resonance structures of the delocalized electrons in the guaiacyl ℼ system.

## 5 Conclusions

In conclusion, we demonstrated that tricin, salcolin A, and salcolin B induce cell death in ATC cell lines by increasing necrosis, and their activity may depend on the origin of the thyroid cell lines. The phytochemical analysis of the dichloromethane phase revealed a chemical profile similar to other species of *Casearia*, suggesting that the shikimate biosynthesis pathway is prominently present.

## Author Contributions

**Augusto L. Santos**: conceptualization, data curation, formal analysis, investigation, methodology, software, writing original draft. **Mariana T. Rodrigues**: conceptualization, data curation, formal analysis, investigation, methodology, software. **Ana Paula Michelli**: data curation, formal analysis, investigation, methodology, software. **Rodrigo E. Tamura**: formal analysis, methodology, writing original draft. **Ileana G. S. de Rubió**: formal analysis, methodology, conceptualization. **Marisi G. Soares**: data curation. **Marcelo J. P. Ferreira**: formal analysis, methodology, funding acquisition, review and editing. **Patricia Sartorelli**: conceptualization, funding acquisition, project administration, visualization, resources, review and editing, supervision.

## Conflicts of Interest

The authors declare no conflicts of interest.

## Supporting information




**Supporting File 1**: cbdv70446‐sup‐0001‐SuppMat.docx

## Data Availability

The authors have nothing to report.
