# Supplementary material for: Exploring the Antiproliferative Activity of Flavolignans From the Leaves of Casearia arborea (Salicaceae)
Source: Chem Biodivers. 2025 Sep 5;22(12):e01489. doi: 10.1002/cbdv.202501489 (PMC12716014; doi:10.1002/cbdv.202501489)

**Exploring the Antiproliferative Activity of Flavolignans**

**from the Leaves of *Casearia arborea* (Salicaceae)**

Augusto L. Santos^†^, Mariana T. Rodrigues^‡^, Ana Paula Micheli^‡^, Rodrigo E. Tamura^†^,

Ileana G. S. de Rubió^†&^, Marisi G. Soares^⁑^, Marcelo J. P. Ferreira^§^, Patricia Sartorelli^†^*

**SUPPLEMENTARY MATERIAL**

| S1 | Spectral family of phenolics and phenylpropanoids. | i |
| --- | --- | --- |
| S2 | Spectral family of flavonoid derivatives. | i |
| S3 | Spectral family of terpenoid derivatives. | ii |
| S4 | Spectral family of fatty acetate derivatives. | ii |
| S5 | Salcolin - HR-ESI-(+)-MS/MS | iii |
| S6 | Salcolin - HR-ESI-(-)-MS/MS | iii |
| S7 | Salcolin - NOESY | iv |

**Supplementary Material S1:** Spectral family of phenolics and phenylpropanoids.

**
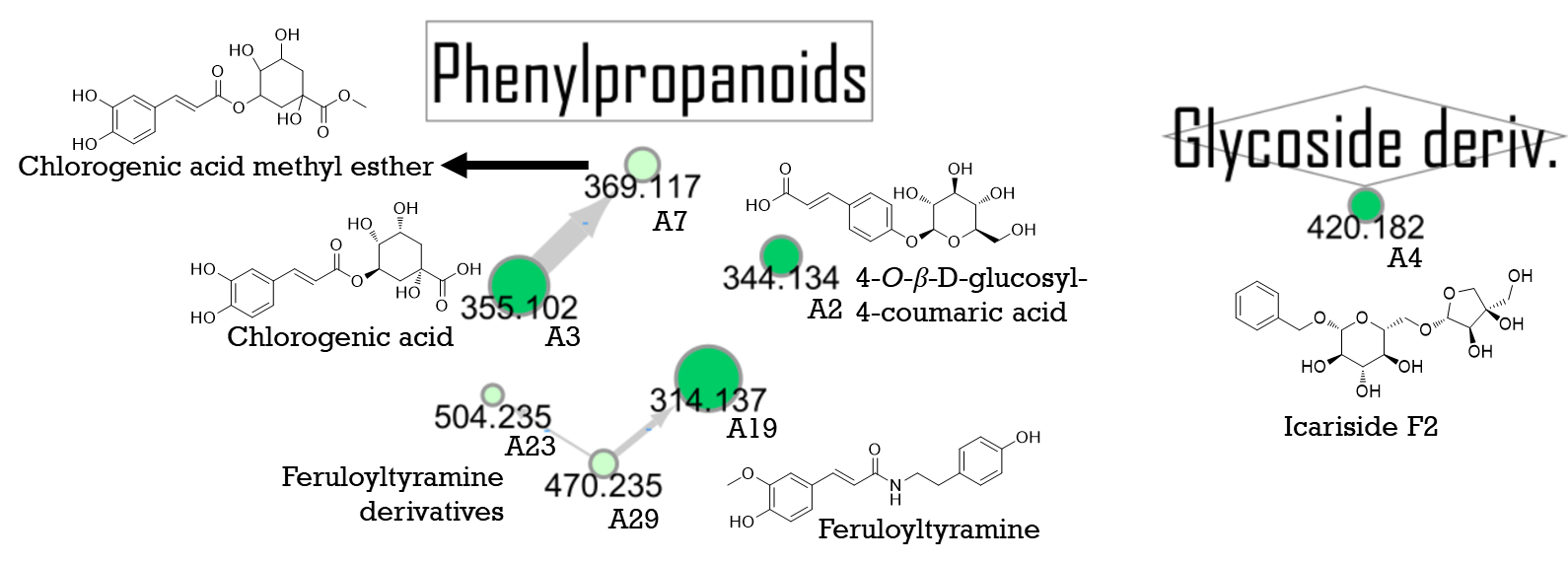
**

**Supplementary Material S2:** Spectral family of flavonoid derivatives.


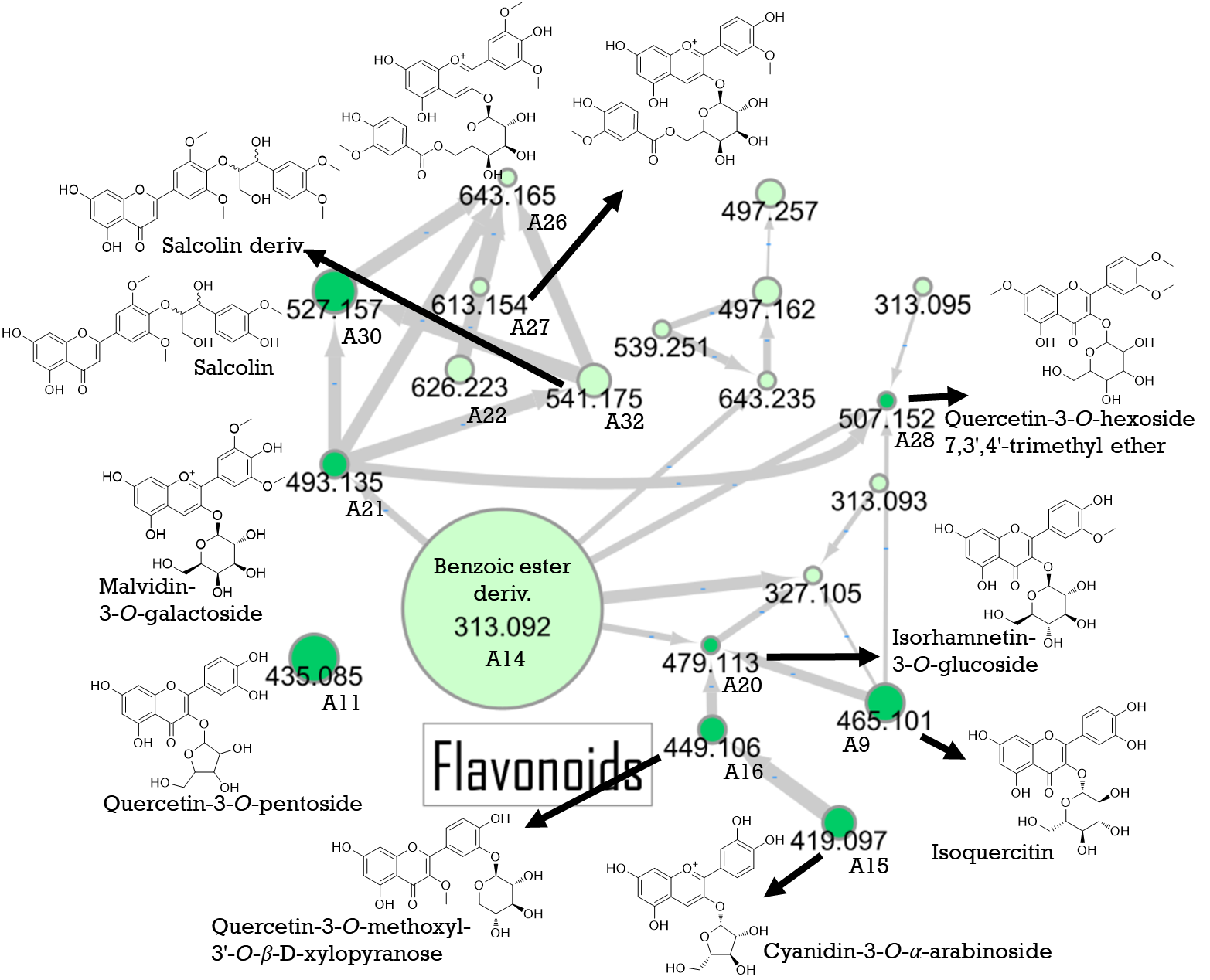


**Supplementary Material S3:** Spectral family of terpenoid derivatives.


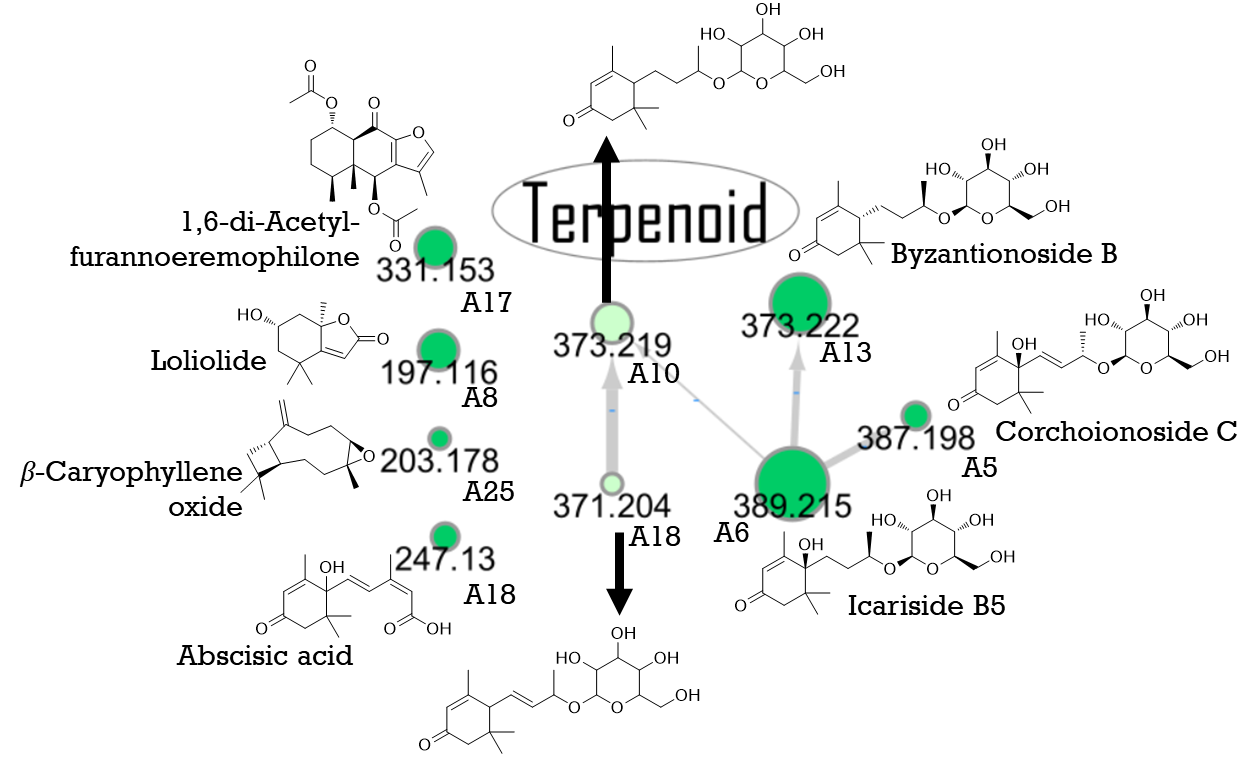


**Supplementary Material S4:** Spectral family of fatty acetate derivatives.


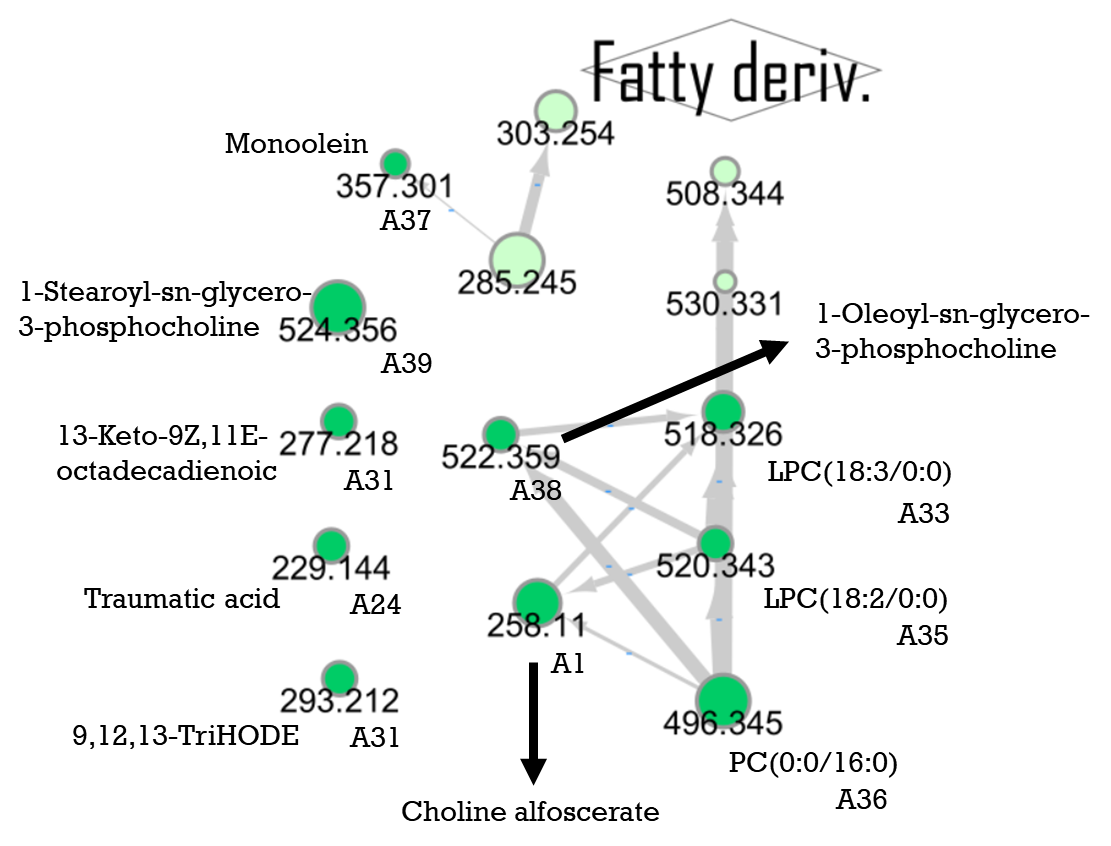


**Supplementary Material S5:** Salcolin - HR-ESI-(+)-MS/MS.


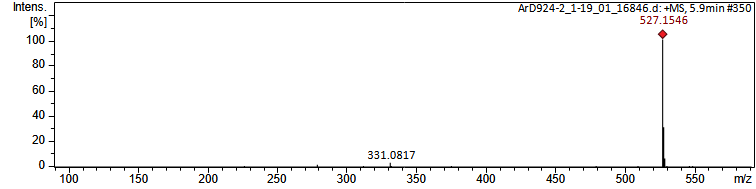


**Supplementary Material S6:** Salcolin - HR-ESI-(-)-MS/MS.


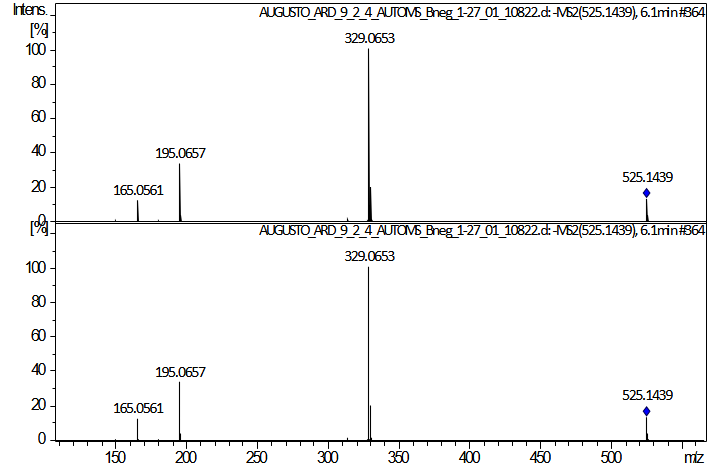


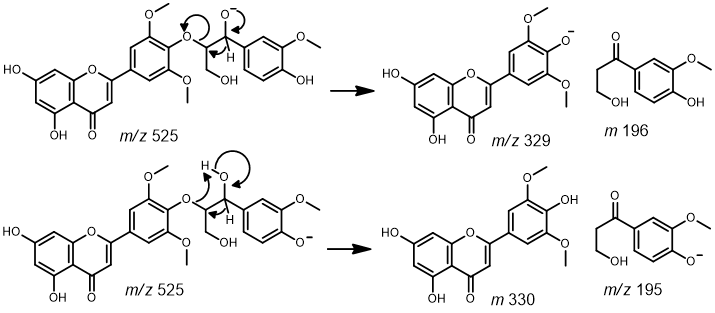


**Supplementary Material S7:** Salcolin - NOESY


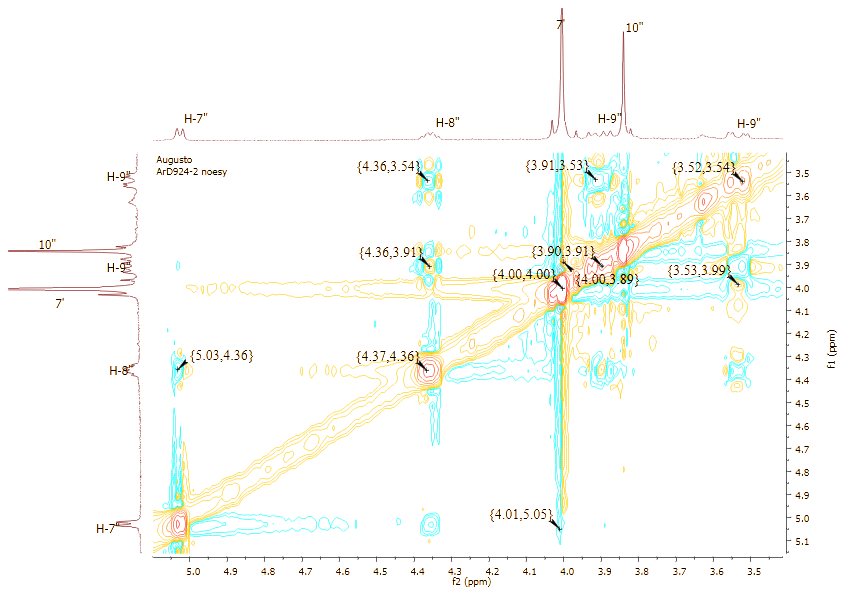

Supplement: Supplementary file 1 — Supporting File 1: cbdv70446‐sup‐0001‐SuppMat.docx [file CBDV-22-e01489-s001.docx]
